# Analysis of factors influencing HPV vaccination willingness of parents of students in China based on the theory of planned behavior

**DOI:** 10.3389/fpubh.2026.1898825

**Published:** 2026-09-16

**Authors:** Yue Li, Jiaqi Li, Xiuhua Sun, Xia Li, Jingli Wu, Congying Li, Congcong Jiao, Wenming Cao, Ya Liu

**Affiliations:** 1 Department of Gynecology, Shandong First Medical University Affiliated Heze Hospital (Heze Municipal Hospital), Heze, Shandong, China; 2 Heze Municipal Center for Disease Control and Prevention, Heze, Shandong, China; 3 Department of Gynecology, Pingshan District Central Hospital of Shenzhen, Shenzhen, Guangdong, China

**Keywords:** children health, human papillomavirus, theory of planned behavior, vaccine, willingness

## Abstract

**Purpose:**

To examine HPV vaccination willingness and its associated factors among parents of female primary and secondary school students in China, and to provide evidence for improving local HPV vaccination coverage.

**Methods:**

This cross-sectional study recruited parents of female primary and secondary school students in Heze City using stratified sampling. A TPB-based questionnaire was administered through an online self-administered survey. Multivariable logistic regression and structural equation modeling were used to examine factors associated with HPV vaccination willingness and behavioral intention.

**Results:**

A total of 506 parents of female primary and secondary school students were included in this cross-sectional study. Among them, 320 parents (63.24%) reported that they were willing to arrange HPV vaccination for their daughters, whereas the reported actual vaccination uptake was 17.98%. Multivariable logistic regression analysis showed that higher parental education, a monthly household income of RMB 5,000–10,000, a family history of cancer, and higher attitude toward the behavior, subjective norm, and behavioral intention scores were associated with greater HPV vaccination willingness. In the TPB structural equation model, attitude toward the behavior (standardized path coefficient = 0.49, *p* < 0.01) and subjective norm (standardized path coefficient = 0.25, *p* < 0.01) were positively associated with behavioral intention, whereas perceived behavioral control showed a significant negative path coefficient (β = −0.10, *p* < 0.01). This negative association was interpreted in light of the fact that the PBC items primarily measured perceived barriers rather than facilitating control.

**Conclusion:**

Parents of female primary and secondary school students in Heze City have a moderate willingness to vaccinate against HPV, but the actual vaccination rate is low.

## 1 Introduction

Human papillomavirus (HPV) is a double-stranded DNA virus belonging to the papillomavirus family. There are currently over 200 known types of HPV (1). Based on their pathogenicity and oncogenic potential, HPV types are classified as high-risk (HR-HPV) and low-risk (LR-HPV). High-risk types include HPV-33, HPV-58, HPV-16, HPV-18, etc. (2). Low-risk types include HPV-6, HPV-11, etc. HPV causes lesions by infecting human skin or mucosal epithelial cells. Approximately 80% of women will be infected at least once in their lifetime. Most low-risk and some high-risk HPV infections are self-limiting and can resolve on their own through immune response within a certain period. However, a portion of women become persistent carriers. Long-term HPV infection can lead to the development of related malignant diseases (3, 4).

Cervical cancer is a common malignant tumor that harms women’s health and is a global public health issue threatening human health. 99.7% of cervical cancer cases are associated with persistent HPV infection, particularly high-risk HPV infection. HR-HPV DNA is found in over 90% of cervical cancer cases, with HPV-16 and HPV-18 being the most common subtypes leading to cervical cancer. Cervical cancer is now the fourth most common cancer among women worldwide and the fourth leading cause of cancer death in women (5). In recent years, the incidence and mortality rates of cervical cancer in China have been increasing annually (5). Besides cervical cancer, HPV infection has also been proven to be associated with the occurrence of diseases such as oropharyngeal cancer. Approximately 70% of oropharyngeal squamous cell carcinomas are caused by HPV infection, while low-risk HPV infection can lead to genital warts (6, 7).

Clinical evidence shows that HPV vaccines can effectively prevent HPV infection and the occurrence of cervical cancer. Vaccination, as primary prevention, can directly block HPV infection at the source, thereby controlling the occurrence of cervical cancer and precancerous lesions, which is crucial for eliminating cervical cancer. The average age of first sexual intercourse among Chinese women is around 16 years old. Therefore, school-aged girls are a key target population for HPV vaccination in China, and increasing the HPV vaccination coverage rate among primary and secondary school girls is of great significance. Since 2020, China has launched HPV vaccination pilot programs in multiple cities, providing free vaccines or vaccination subsidies for school-aged girls. However, the willingness and attitudes of eligible girls’ parents towards this government initiative remain uncertain. Psychological factors have been considered by many scholars as the best explanation for vaccine hesitancy. Parents’ attitudes toward HPV vaccination are an important factor associated with vaccination intention.

The Theory of Planned Behavior (TPB) is a representative theory in social psychology concerning the relationship between attitudes and behavior (8). It posits that attitude toward the behavior, subjective norm, and perceived behavioral control are three interrelated factors influencing an individual’s behavioral intention, which is the most direct factor affecting actual behavior. Due to its good explanatory power, consistency, and authority in exploring the mechanisms of behavioral intention, this theory is widely applied in health behavior research.

This study takes this as a starting point. Using the questionnaire-design framework of the Theory of Planned Behavior (TPB), we conducted a questionnaire survey among parents of female primary and secondary school students in Heze City. Structural equation modelling was adopted to validate this theoretical framework. We examined the associations between the TPB constructs and HPV vaccination willingness and behavioral intention. The findings can provide references for governments and medical institutions to optimise vaccination services, reduce HPV vaccine hesitancy, improve vaccination coverage, and achieve effective cervical cancer prevention.

## 2 Methods

### 2.1 Research objects

The participants were parents of primary and secondary students aged 9–14 residing in counties and districts of Heze City.

Inclusion criteria: (1) Parents or legal guardians of girls aged 9–14 years; (2) Having normal cognitive ability and capable of independently completing the online questionnaire; (3) Voluntary participation in this study. Exclusion criteria: Guardians suffering from severe mental illness or cognitive impairment.

### 2.2 Research methods

A cross-sectional survey was conducted among parents of primary and secondary school students aged 9–14 years in counties and districts of Heze City. Stratified sampling was adopted, and the detailed sampling procedures were as follows:

Several counties and districts in Heze City were selected as survey sites. With the assistance of local primary and secondary schools, WeChat groups for student families in these areas were contacted.

Researchers distributed electronic questionnaire links in these WeChat groups. The opening page of the questionnaire clarified the research objectives, survey contents, anonymity principle, confidentiality requirements and the principle of voluntary participation.

Potential participants judged their eligibility according to the inclusion and exclusion criteria. Eligible guardians who provided informed consent took part in the survey and completed the online questionnaire independently.

After data collection, data cleaning was performed. Questionnaires with substantial missing values, repeated submissions and obvious logical contradictions were excluded, and valid questionnaires were retained for subsequent statistical analysis.

After excluding data that did not meet the inclusion criteria, the final sample size for this study was 506 participants. The formal survey period spanned from October 1, 2022, to March 30, 2023.

### 2.3 Ethics approval and informed consent

The study protocol, investigation tools, subject inclusion criteria and intervention procedures were reviewed by Ethics Committee of Heze Municipal Hospital. (Ethics approval No.: 2024-KY003-06), and the approval documents are fully archived. The ethics committee comprehensively evaluated the potential risks and subject protection measures of this study and approved its implementation. The whole research was carried out in strict accordance with the approved protocol. Any subsequent protocol amendments will be submitted to the ethics committee for re-review, and written approval shall be obtained prior to implementation.

All subjects were fully informed of the research purpose, procedures, participation duration, potential benefits, foreseeable risks, data collection methods and information storage regulations. Researchers provided information in plain language to ensure subjects had sufficient time to make autonomous decisions. Subjects could voluntarily participate and unconditionally withdraw from the study at any stage without any adverse consequences. Specialized information notification procedures and targeted protection mechanisms were adopted for vulnerable populations. Written informed consent was obtained from all subjects before enrollment. Electronic informed consent adopted under special circumstances was approved by the ethics committee, and a copy of the consent document was provided to each subject.

This study was conducted in accordance with the principles of the Declaration of Helsinki and international ethical standards for biomedical research involving human participants. All personal information was de-identified. Research data were only used for analysis in this project and shall not be disclosed or used for other purposes. Risk prevention plans were established for information collection, sample acquisition and intervention operations to minimize the burden and potential harm to subjects, balancing scientific value and the protection of subjects’ rights and interests.

There is no conflict of interest in this study, and no commercial institution interferes with the research. Raw data are stored with encryption and hierarchical access control. No personally identifiable information of subjects will be disclosed in publications.

### 2.4 Research content

The questionnaire comprised two parts. The first collected sociodemographic information, including parental age, residence, occupation, education level, monthly household income, family medical history, and child health-related characteristics. The second assessed HPV vaccination willingness and four TPB-related constructs. In this study, HPV vaccination willingness was defined as a parent’s general inclination to arrange HPV vaccination for their daughter, whereas behavioral intention represented a more action-oriented plan or motivation to do so. The English translation of the Chinese questionnaire is provided in Supplementary File S1.

*Vaccination willingness (1 item)*: I am willing to arrange HPV vaccination for my daughter. It represents the general subjective acceptance, reflecting only a vague subjective inclination without concrete action-oriented plans.

*Attitude toward the behavior*: Individuals’overall positive or negative evaluation of taking-up vaccination. Higher scores indicate more favorable attitudes toward vaccination. (4 items: I believe HPV vaccination will reduce the risk of future HPV infection and cervical cancer; I believe HPV vaccination is beneficial to others; I believe it is necessary to get the HPV vaccine; I believe it is necessary to obtain the HPV vaccine).

*Subjective norm*: Perceived social pressure from significant referents (family, friends, healthcare workers) regarding whether one should get vaccinated, together with motivation to comply with these expectations. Higher scores indicate stronger perceived social normative pressure. (3 items: If relatives and friends get vaccinated, I will also let my child get vaccinated; If community government staff recommend vaccination, I will also let my child get vaccinated; If a doctor advises me to vaccinate, I will let my child get vaccinated).

*Perceived behavioral control (PBC)*: Perceived ease or difficulty of performing vaccination, including self-assessed ability, available resources, opportunities and external barriers. Note that several scale items focused on behavioral barriers; higher scores correspond to greater perceived obstacles and lower actual perceived controllability. (6 items: The high cost of the vaccine affects my willingness to vaccinate my child; Side effects of the vaccine; Child’s age; Vaccine; Complexity of HPV vaccination; Uncertainty about domestic HPV vaccines being inferior to imported ones).

*Behavioral intention*: It refers to individuals’ subjective willingness, plans and motivations for future vaccination, representing an action-oriented tendency with concrete intentions and plans. As a proximal latent variable in the Theory of Planned Behavior (TPB), it is measured by multi-item scale. (3 items: I will consider vaccinating my child with the HPV vaccine; I will vaccinate my child with the HPV vaccine in the future; I will recommend the HPV vaccine to relatives and friends), along with a section on actual execution behavior. Behavioral Intention served as the outcome variable in this study. Notably, the PBC items in this study primarily reflect perceived barriers to vaccination (e.g., cost, side effects, and accessibility); therefore, higher PBC scores indicate greater perceived obstacles, which is expected to negatively affect Behavioral Intention.

In the TPB framework, attitude toward the behavior, subjective norm, and perceived behavioral control are theorized to be associated with behavioral intention, which is a proximal determinant of subsequent behavior. Because this study was cross-sectional, these relationships were interpreted as associations rather than causal effects. The questionnaire structure and the operationalization of the TPB constructs are shown in Figure 1.

**Figure 1:**
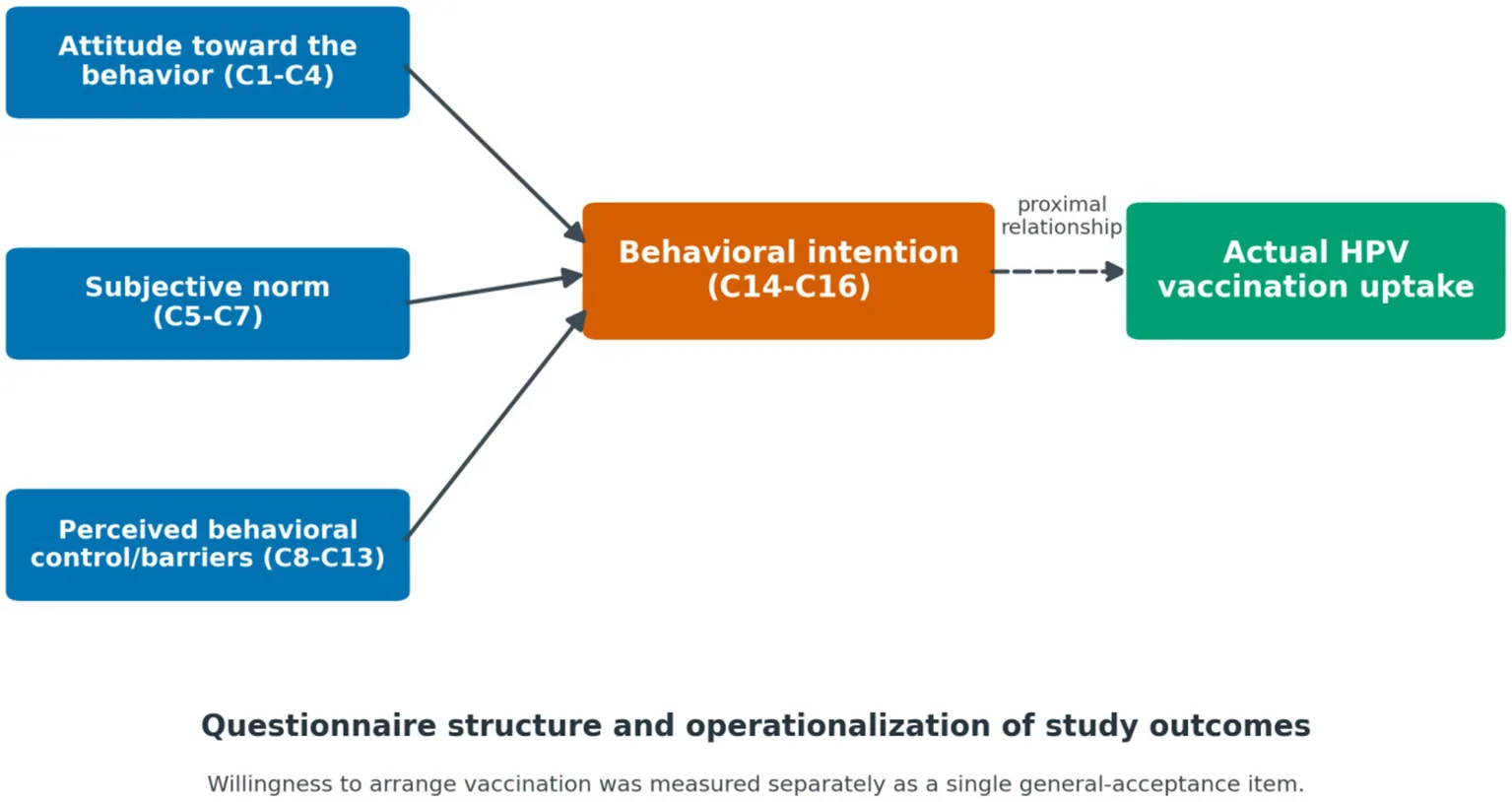
Questionnaire structure and operationalization of the TPB constructs. C1-C4 assessed attitude toward the behavior, C5-C7 assessed subjective norm, C8-C13 assessed perceived behavioral control/barriers, and C14-C16 assessed behavioral intention. Willingness to arrange vaccination was measured separately as a single item, and actual HPV vaccination uptake was assessed separately.

### 2.5 Scale development and quality control

Scale development: The scale in this study was developed based on the Theory of Planned Behavior (TPB) (9). According to this theory, behavioral intention serves as the most direct predictor of actual behavior, which is jointly affected by three core constructs: attitude toward the behavior, subjective norm, and perceived behavioral control. The scale was developed following standard procedures. Firstly, salient beliefs were extracted from open-ended preliminary surveys among the target population to generate an initial item pool. Secondly, items were screened through Delphi expert consultation. Finally, item screening as well as reliability and validity tests were performed using pre-survey data.

The questionnaire was used after adjustments based on a pre-survey. The sample demonstrated good reliability (Cronbach’s *α* = 0.781) and validity (KMO = 0.804, *p* < 0.001). All investigators received professional systematic training before the survey. After questionnaire collection, dedicated personnel reviewed them. Any errors or omissions were addressed by re-conducting the survey. All questionnaires underwent double data entry and logical verification. Questionnaires that did not meet requirements were excluded.

### 2.6 Statistical processing

Excel 2021 was used for data organization, logical verification, and database construction. Statistical analyses were performed using SPSS version 26.0, and structural equation modeling (SEM) was conducted using AMOS version 28.0. Categorical variables were summarized as frequencies and percentages, whereas continuous variables were summarized as means and standard deviations. All statistical tests were two-sided, and a *p*-value < 0.05 was considered statistically significant.

In the univariable analyses, the chi-square (*χ*^2^) test was used to compare categorical variables between the willing and unwilling groups, and independent-samples t-tests were used to compare TPB component scores. For reliability analysis, Cronbach’s *α* ≥ 0.70 and a corrected item-total correlation (CITC) ≥ 0.40 were considered acceptable. An item was considered for removal if its deletion resulted in a meaningful increase in Cronbach’s α. Exploratory factor analysis (EFA) was conducted to assess the construct structure. A Kaiser–Meyer–Olkin (KMO) value ≥ 0.70 and a significant Bartlett’s test of sphericity were considered acceptable prerequisites for factor analysis. Factors with eigenvalues >1 were retained, and factor loadings ≥0.50 were considered acceptable for item attribution.

For the multivariable logistic regression analysis, the dependent variable was parental willingness to arrange HPV vaccination for their daughters, coded as 1 for willing and 0 for unwilling. Candidate covariates were selected based on the pre-specified study framework and variables showing statistically significant differences in the univariable comparisons. The final model included parental education level, monthly household income, family history of cancer, and the dichotomized TPB component scores. For each TPB component, scores greater than the corresponding median were coded as 1, whereas scores equal to or below the median were coded as 0. The selected variables were entered into the multivariable logistic regression model, and adjusted odds ratios (ORs) with 95% confidence intervals (CIs) were estimated. The reference categories were primary or junior high school education or below, a monthly household income below RMB 5,000, no family history of cancer, and TPB scores equal to or below the corresponding cut-off values.

Records with substantial missing data, duplicate submissions, or obvious logical inconsistencies were excluded during the pre-specified data-cleaning process before statistical analysis. The number of observations included in each analysis was reported where applicable.

Guided by the Theory of Planned Behavior (TPB), an SEM was constructed with attitude toward the behavior, subjective norm, and perceived behavioral control as exogenous latent variables and behavioral intention as the endogenous latent variable. Because the perceived behavioral control items primarily assessed perceived barriers to vaccination, higher scores were interpreted as indicating stronger perceived barriers rather than greater facilitating control. The maximum likelihood (ML) method was used for parameter estimation. After evaluation of the measurement model, path analysis was conducted to estimate the standardized path coefficients (β) of the latent variables in relation to behavioral intention. Model fit was evaluated using the *χ*^2^/df ratio, comparative fit index (CFI), Tucker–Lewis index (TLI), root mean square error of approximation (RMSEA), and standardized root mean square residual (SRMR). A *p*-value < 0.05 was considered statistically significant.

The following hypotheses were proposed:

*H1*: Attitude toward the behavior is positively associated with behavioral intention.

*H2*: Subjective norm is positively associated with behavioral intention.

*H3*: Perceived behavioral control is positively associated with behavioral intention.

Because this study used a cross-sectional design, the regression estimates and SEM path coefficients were interpreted as statistical associations rather than evidence of causal relationships.

## 3 Results

### 3.1 Participant characteristics and group comparisons

Among the 506 participating parents, 91 reported that their daughters had received HPV vaccination, corresponding to an actual vaccination uptake of 17.98%. In addition, 320 parents (63.24%) reported that they were willing to arrange HPV vaccination for their daughters, whereas 186 (36.76%) were unwilling. Most of the daughters were attending junior high school (411/506, 81.23%), and most parents had rural household registration (479/506, 94.66%). The largest age group was 35–45 years (347/506, 68.58%). Most families reported a monthly household income below RMB 5,000 (343/506, 67.79%), and most parents had a primary or junior high school education or below (369/506, 72.92%).

No statistically significant differences were observed between the willing and unwilling groups in the child’s educational stage, household registration, parent’s age, occupation type, work busyness, or insurance type (all *p* > 0.05). Significant between-group differences were observed in parental education level, monthly household income, and family history of cancer (all *p* ≤ 0.05). Compared with the unwilling group, the willing group included higher proportions of parents with a university education or above, a monthly household income of RMB 5,000–10,000 or above RMB 10,000, and a family history of cancer. Detailed results are presented in Table 1. The difference between reported willingness and actual HPV vaccination uptake is illustrated in Figure 2.

**Table 1:** Comparison of demographic characteristics of parents with different HPV vaccination willingness.

Characteristic	Category	Willing group, *n* (%)	Unwilling group, *n* (%)	*χ* ^2^	*p*-value
Child’s educational stage	Primary school	57 (17.81)	38 (20.43)	0.53	0.47
	Junior high school	263 (82.19)	148 (79.57)		
Household registration	Urban	16 (5.00)	11 (5.91)	0.20	0.66
	Rural	304 (95.00)	175 (94.09)		
Parent’s age	<35 years	39 (12.19)	23 (12.37)	0.33	0.85
	35–45 years	222 (69.38)	125 (67.20)		
	>45 years	59 (18.44)	38 (20.43)		
Parent’s education level	University or above	30 (9.38)	6 (3.23)	9.15	0.01
	Technical secondary school or high school	69 (21.56)	32 (17.20)		
	Primary or junior high school or below	221 (69.06)	148 (79.57)		
Occupation type	Housework or farming	239 (74.69)	153 (82.26)	4.05	0.13
	Worker or self-employed person	74 (23.13)	31 (16.67)		
	Public officer or technical professional	7 (2.19)	2 (1.08)		
Work busyness	Very busy	48 (15.00)	31 (16.67)	0.66	0.72
	Moderately busy	260 (81.25)	146 (78.49)		
	Not busy	12 (3.75)	9 (4.84)		
Monthly household income, RMB	<5,000	199 (62.19)	144 (77.42)	15.71	<0.001
	5,000–10,000	85 (26.56)	36 (19.35)		
	>10,000	36 (11.25)	6 (3.23)		
Insurance type	No insurance	51 (15.94)	35 (18.82)	0.69	0.41
	Social or commercial insurance	269 (84.06)	151 (81.18)		
Family history of cancer	Yes	47 (14.69)	13 (6.99)	6.67	0.01
	No	273 (85.31)	173 (93.01)		

Data are presented as *n* (%), with percentages retained to two decimal places to maintain consistency with the reported frequency data. HPV, human papillomavirus. The willing group comprised parents who reported that they were willing to arrange HPV vaccination for their daughters, whereas the unwilling group comprised parents who reported that they were unwilling. A two-sided *p* < 0.05 was considered statistically significant.

**Figure 2:**
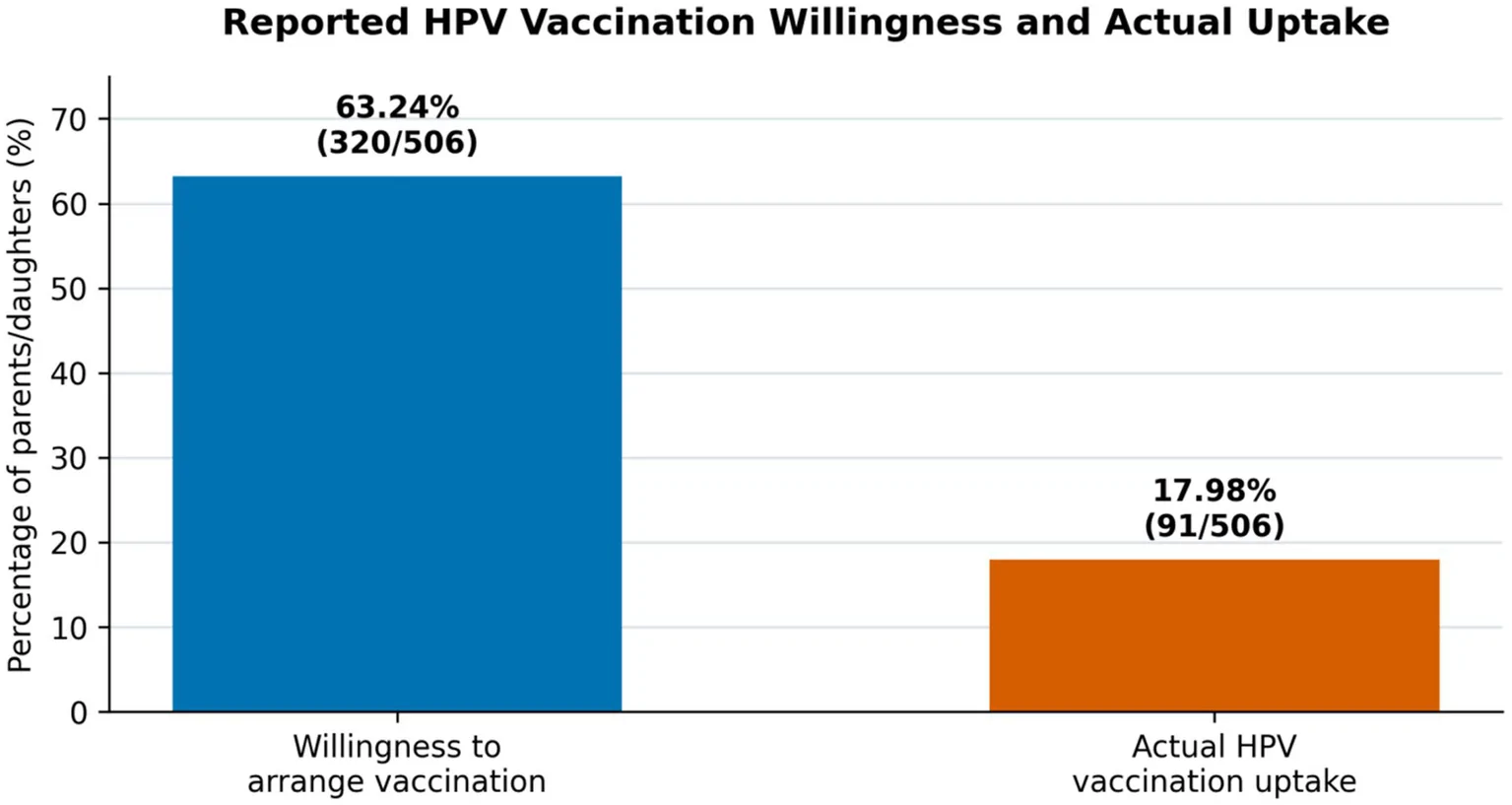
Reported HPV vaccination willingness and actual uptake. Willingness was 320/506 (63.24%), and actual HPV vaccination uptake was 91/506 (17.98%).

### 3.2 Reliability and validity of the TPB scale

The Cronbach’s α coefficients for the four TPB dimensions ranged from 0.721 to 0.773, indicating acceptable internal consistency. Item factor loadings ranged from 0.558 to 0.821, and the KMO value was 0.804. Most corrected item-total correlation (CITC) values were ≥0.40, except for item C13, which had a CITC value of 0.341. Because deleting C13 resulted in only a limited increase in Cronbach’s α and the item had a relatively high factor loading, it was retained. These findings supported the internal consistency and construct validity of the TPB scale. See Table 2.

**Table 2:** Reliability and validity analysis of the TPB model for parents’ HPV vaccination willingness.

Dimension	Item	Mean	SD	Factor loading	CITC	Cronbach’s α if item deleted	Cronbach’s α	KMO
Attitude toward the behavior	C1. HPV vaccination can effectively prevent HPV infection and cervical cancer	3.54	0.744	0.699	0.546	0.655	0.727	0.804
	C2. My child needs HPV vaccination	3.28	0.935	0.703	0.591	0.619		
	C3. I would feel reassured after my child receives HPV vaccination	3.06	0.891	0.708	0.545	0.648		
	C4. HPV vaccination is beneficial to people in the target age group	3.34	0.853	0.633	0.400	0.731		
Subjective norm	C5. If relatives or friends recommend HPV vaccination, I will vaccinate my child	3.02	0.917	0.683	0.500	0.682	0.721	0.804
	C6. If a doctor recommends HPV vaccination, I will vaccinate my child	3.46	0.995	0.712	0.588	0.575		
	C7. If the government or community recommends HPV vaccination, I will vaccinate my child	3.20	1.103	0.639	0.547	0.633		
Perceived behavioral control	C8. The complexity of treating HPV infection would affect my decision	3.55	0.972	0.558	0.474	0.715	0.746	0.804
	C9. The high cost of HPV vaccination would affect my decision	2.88	0.893	0.575	0.512	0.702		
	C10. Vaccine-related side effects would affect my decision	3.13	0.918	0.662	0.559	0.688		
	C11. The effectiveness of the HPV vaccine would affect my decision	3.41	0.778	0.656	0.456	0.718		
	C12. Whether my child was of an appropriate age for vaccination would affect my decision	3.01	0.801	0.606	0.572	0.687		
	C13. Uncertainty about the quality of domestically produced HPV vaccines would affect my decision	2.82	0.741	0.821	0.341	0.745		
Behavioral intention	C14. I will recommend HPV vaccination to relatives and friends	3.62	0.711	0.743	0.575	0.759	0.773	0.804
	C15. I will consider arranging HPV vaccination for my child	3.55	0.947	0.759	0.689	0.603		
	C16. I will arrange HPV vaccination for my daughter in the future	3.16	1.184	0.652	0.642	0.697		

TPB, Theory of Planned Behavior; HPV, human papillomavirus; SD, standard deviation; CITC, corrected item-total correlation; KMO, Kaiser–Meyer–Olkin measure. Items were scored using a five-point Likert scale, with higher scores indicating stronger agreement. Cronbach’s *α* ≥ 0.70, CITC ≥0.40, and factor loading ≥0.50 were considered acceptable. The KMO value of 0.804 represented the overall scale-level measure.

### 3.3 Comparison of TPB component scores

Independent-samples t-tests showed that parents in the willing group had higher scores than those in the unwilling group for attitude toward the behavior, subjective norm, perceived behavioral control, and behavioral intention. All between-group differences were statistically significant (all *p* < 0.001; Table 3).

**Table 3:** Comparison of TPB component scores between HPV vaccination willingness groups.

TPB component	Willing group (*N* = 320), M ± SD	Unwilling group (*N* = 186), M ± SD	*t*-value	*P*-value
Attitude toward the behavior	13.94 ± 2.43	11.98 ± 2.26	8.99	<0.001
Subjective norm	10.34 ± 2.17	8.54 ± 2.41	8.63	<0.001
Perceived behavioral control	19.36 ± 2.95	17.81 ± 3.88	5.08	<0.001
Behavioral intention	11.13 ± 2.23	9.06 ± 2.61	9.46	<0.001

TPB, Theory of Planned Behavior; M, mean; SD, standard deviation. Scores were calculated by summing the items within each dimension. Independent-samples t-tests were used for between-group comparisons. Because the PBC items primarily assessed perceived barriers rather than facilitating control, higher PBC scores should not be interpreted as greater actual control over vaccination.

### 3.4 Correlations among TPB components and behavioral intention

Pearson correlation analysis showed that attitude toward the behavior was positively correlated with subjective norm (*r* = 0.31, *p* < 0.001), perceived behavioral control (*r* = 0.10, *p* < 0.05), and behavioral intention (*r* = 0.42, *p* < 0.001). Subjective norm was positively correlated with perceived behavioral control (*r* = 0.20, *p* < 0.001) and behavioral intention (*r* = 0.35, *p* < 0.001). However, the correlation between perceived behavioral control and behavioral intention was weak and not statistically significant (*r* = 0.03, *p* = 0.48). See Table 4.

**Table 4:** Pearson correlations among TPB component scores and behavioral intention.

Variable	Statistic	Attitude toward the behavior	Subjective norm	Perceived behavioral control	Behavioral intention
Attitude toward the behavior	Pearson’s r	1.00	0.31	0.10	0.42
	*p* value	—	<0.001	<0.05	<0.001
Subjective norm	Pearson’s r	0.31	1.00	0.20	0.35
	*P* value	<0.001	—	<0.001	<0.001
Perceived behavioral control	Pearson’s r	0.10	0.20	1.00	0.03
	*P* value	<0.05	<0.001	—	0.48
Behavioral intention	Pearson’s r	0.42	0.35	0.03	1.00
	*P* value	<0.001	<0.001	0.48	—

TPB, Theory of planned behavior; HPV, human papillomavirus. Correlations were evaluated using Pearson’s correlation analysis. The diagonal represents the correlation of each variable with itself.

### 3.5 Factors associated with HPV vaccination willingness

Parental education level, monthly household income, and family history of cancer, which showed statistically significant between-group differences in the univariate analysis, together with the dichotomized TPB component scores, were included as independent variables in the multivariable logistic regression model. Higher parental education, a monthly household income of RMB 5,000–10,000, a family history of cancer, and higher scores for attitude toward the behavior, subjective norm, and behavioral intention were associated with greater HPV vaccination willingness. Compared with parents with a primary or junior high school education or below, parents with a university education or above had higher odds of reporting HPV vaccination willingness (OR = 3.07, 95% CI: 1.09–8.66, *p* = 0.03). Compared with families with a monthly income below RMB 5,000, families with a monthly income of RMB 5,000–10,000 had higher odds of reporting vaccination willingness (OR = 3.56, 95% CI: 1.32–9.63, *p* = 0.01). The association for a monthly income above RMB 10,000 was not statistically significant (OR = 1.81, 95% CI: 0.63–5.22, *p* = 0.27). Parents with a family history of cancer also had higher odds of reporting vaccination willingness (OR = 2.30, 95% CI: 1.11–4.76, *p* = 0.03). Higher attitude toward the behavior scores, subjective norm scores, and behavioral intention scores were significantly associated with greater vaccination willingness. Perceived behavioral control was not statistically significant in the logistic regression model (OR = 1.20, 95% CI: 0.78–1.85, *p* = 0.41). See Tables 5 and 6.

**Table 5:** Coding of variables included in the logistic regression model.

Variable	Coding
Parental education level	0 = Primary or junior high school or below; 1 = Technical secondary school or high school; 2 = University or above
Monthly household income	0 = <RMB 5,000; 1 = RMB 5,000–10,000; 2 = > RMB 10,000
Family history of cancer	0 = No; 1 = Yes
Attitude toward the behavior	0 = TPB score ≤13; 1 = TPB score >13
Subjective norm	0 = TPB score ≤10; 1 = TPB score >10
Perceived behavioral control	0 = TPB score ≤19; 1 = TPB score >19
Behavioral intention	0 = TPB score ≤10; 1 = TPB score >10

TPB, Theory of Planned Behavior. For the TPB dimensions, scores greater than the corresponding median were coded as 1, whereas scores equal to or below the corresponding median were coded as 0. The dependent variable was parental HPV vaccination willingness, coded as 1 for willing and 0 for unwilling.

**Table 6:** Multivariable logistic regression analysis of factors associated with HPV vaccination willingness.

Variable	Category	*n*	*β*	SE	Wald *χ*^2^	*p*-value	OR	95% CI
Parental education level	Primary or junior high school or below	369	—	—	—	—	—	—
	Technical secondary school or high school	101	0.93	0.57	2.68	0.10	2.54	0.83–7.78
	University or above	36	1.12	0.53	4.50	0.03	3.07	1.09–8.66
Monthly household income	<RMB 5,000 (Reference)	343	—	—	—	—	—	—
	RMB 5,000–10,000	121	1.27	0.51	6.26	0.01	3.56	1.32–9.63
	>RMB 10,000	42	0.59	0.54	1.20	0.27	1.81	0.63–5.22
Family history of cancer	No (Reference)	446	—	—	—	—	—	—
	Yes	60	0.83	0.37	5.03	0.03	2.30	1.11–4.76
Attitude toward the behavior	TPB score ≤13 (Reference)	287	—	—	—	—	—	—
	TPB score >13	219	1.30	0.25	28.02	<0.001	3.68	2.27–5.96
Subjective norm	TPB score ≤10 (Reference)	312	—	—	—	—	—	—
	TPB score >10	196	0.79	0.24	10.60	<0.001	2.20	1.37–3.53
Perceived behavioral control	TPB score ≤19 (Reference)	288	—	—	—	—	—	—
	TPB score >19	218	0.18	0.22	0.68	0.41	1.20	0.78–1.85
Behavioral intention	TPB score ≤10 (Reference)	257	—	—	—	—	—	—
	TPB score >10	249	1.20	0.24	24.81	<0.001	3.32	2.07–5.33

*β*, regression coefficient; SE, standard error; OR, odds ratio; CI, confidence interval; TPB, Theory of Planned Behavior. Reference categories were primary or junior high school or below for parental education, <RMB 5,000 for monthly household income, no family history of cancer, and scores equal to or below the specified TPB cut-off values. ORs and 95% CIs were estimated using multivariable logistic regression. A two-sided *p* < 0.05 was considered statistically significant.

### 3.6 Structural equation model of behavioral intention

A structural equation model was constructed with attitude toward the behavior, subjective norm, and perceived behavioral control as exogenous latent variables and behavioral intention as the endogenous latent variable. The model showed an acceptable fit to the data, with *χ*^2^/df = 2.86, CFI = 0.92, TLI = 0.90, RMSEA = 0.069 (90% CI: 0.061–0.077), and SRMR = 0.058. The standardized factor loadings of the observed variables ranged from 0.41 to 0.91, indicating that the observed variables adequately reflected their corresponding latent constructs. The standardized error terms were within an acceptable range, indicating that measurement error was adequately controlled. Attitude toward the behavior was positively associated with behavioral intention, with a standardized path coefficient of 0.49 (*p* < 0.01). Subjective norm was also positively associated with behavioral intention, with a standardized path coefficient of 0.25 (*p* < 0.01). The standardized path coefficient for attitude toward the behavior was greater than that for subjective norm. Perceived behavioral control showed a significant negative path coefficient for behavioral intention (*β* = −0.10, *p* < 0.01). This negative coefficient should be interpreted in light of the item wording. The PBC items primarily assessed perceived barriers, including vaccine cost, side effects, child age, treatment complexity, and uncertainty about the quality of domestically produced vaccines. Therefore, higher PBC scores represented stronger perceived barriers rather than greater facilitating control. H1 and H2 were supported, whereas H3 was not supported. Because the study used a cross-sectional design, the standardized path coefficients were interpreted as statistical associations within the proposed model rather than evidence of causal effects. The reported structural paths are presented in Figure 3.

**Figure 3:**
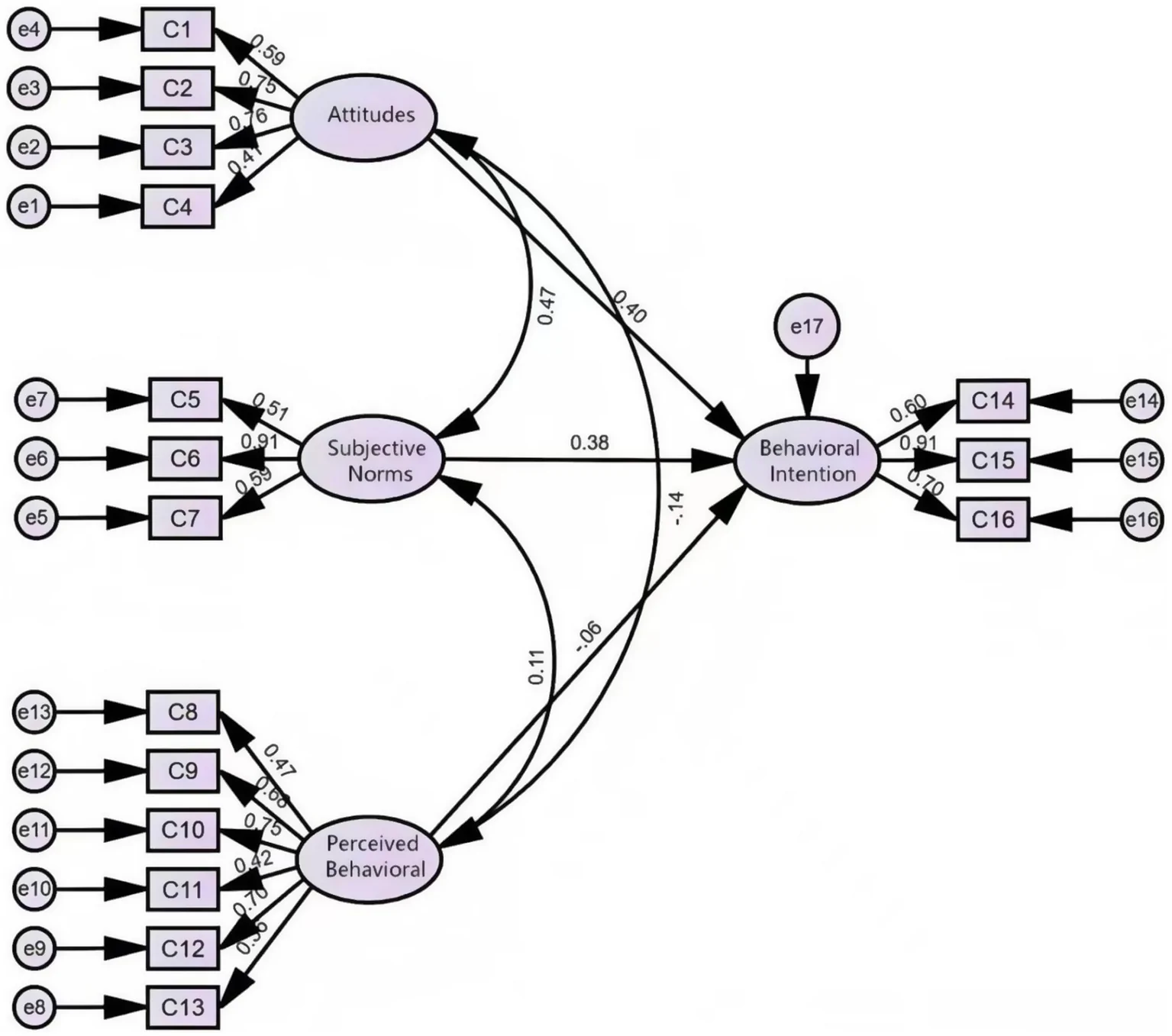
Structural equation model of the associations between TPB constructs and HPV vaccination behavioral intention. TPB, Theory of Planned Behavior; HPV, human papillomavirus. Ellipses represent latent variables, and rectangles represent observed variables. Single-headed arrows indicate hypothesized directional associations, and the values on the arrows represent standardized path coefficients. Double-headed arrows between exogenous latent variables represent correlations. Error terms represent residual variance associated with the observed variables.

## 4 Discussion

Cervical cancer is one of the major malignant tumors threatening women’s health worldwide. In recent years, its incidence has increased year by year, drawing extensive attention due to its high morbidity and mortality as well as the trend of younger onset (10). 99% of cervical cancer cases are associated with persistent infection of high-risk HPV. Vaccination is an effective method to prevent HPV infection and cervical cancer, and cervical cancer has become the only cancer that can be prevented by vaccination so far. The average age of first sexual intercourse among Chinese adolescents is 15.9 years old (11). The World Health Organization recommends that, to effectively prevent cervical cancer, girls aged 9–14 should be prioritized to receive HPV vaccination before the onset of sexual activity, which can achieve better immune effect and protection. All existing global guidelines recommend girls aged 9–14 as the primary or routine vaccination group (12). A survey found that the timing of adolescents’ first sexual intercourse shows an obvious trend of advancing (13). Therefore, early vaccination before adolescents’ first sexual intercourse has become a top priority.

In this study, 506 parents of primary and secondary school students participated in the questionnaire survey. The results showed that the HPV vaccination willingness rate of parents for their children in Heze City was 63.24%, which was comparable to the willingness rate reported in Karamay (61.05%), Shanghai (60.70%), and Tongzhou District of Beijing (71.23%). However, the actual HPV vaccination rate of primary and secondary school girls in this study was only 17.82%, far lower than that in developed countries such as the United States (64%) (14) and Canada (72.3%) (15). A typical gap between high intention and low HPV vaccination uptake is observed in this region, which constitutes a prevalent challenge to HPV vaccine rollout in China and numerous low-and middle-income countries (16, 17). China’s overall HPV vaccination coverage is markedly lower compared with developed countries, leaving a considerable gap to meet the WHO target of 90% vaccination among girls aged under 15 for cervical cancer elimination by 2030 (18).

Critical review of recent global literature shows that high HPV vaccination coverage in developed countries is supported by integrated policies and services: national immunization inclusion, school vaccination, free vaccination, and provider recommendation (19). In China, HPV vaccination remains predominantly self-funded. Vaccination is primarily delivered through outpatient settings, without routine school vaccination programmes for eligible girls aged 9–14 (20). Despite moderately high parental intention, multiple structural barriers, including cost, vaccine availability, access to vaccination services, and online misinformation, may contribute to the gap between intention and vaccination uptake (21). Many regional Chinese studies have focused primarily on parental intention, whereas fewer have examined the factors that may contribute to the intention–behavior gap. Evidence from high-income countries is also difficult to generalize to rural, resource-limited areas such as southwestern Shandong (22). Recent systematic reviews confirm that health promotion alone cannot close the intention-behavior gap, with institutional arrangements and service access playing more dominant roles (16), which underscores the significance of localized evidence generated by this study.

Therefore, this study was designed based on the TPB framework, constructed a structural equation model of HPV behavioral intention, tested the relationship between attitude toward the behavior, subjective norm, perceived behavioral control and behavioral intention, and explored the influencing factors of HPV vaccination willingness among parents of primary and secondary school students. Research shows that parental vaccination attitudes strongly predict vaccination intention; greater positivity in attitudes leads to stronger behavioral intentions. This aligns with recent Chinese HPV vaccine studies adopting the TPB framework (23). In this study, the scores of vaccine safety and necessity were relatively low, indicating that parents lacked sufficient confidence in HPV vaccines. Previous studies have shown that the incidence of cervical cancer is significantly reduced in adolescent girls vaccinated against HPV (24). The Global Advisory Committee on Vaccine Safety, based on regularly reviewed evidence, confirms that the safety of HPV vaccines is guaranteed, and all currently available HPV vaccines have good safety profiles (25). Another previous study found that the incidence of cervical cancer decreased by 97% in adolescent girls who received HPV vaccination; a separate study on adverse reactions after HPV vaccination showed that the vaccine was well tolerated, most of the injection site and systemic adverse reactions were mild to moderate, and no serious adverse events were found to be associated with HPV vaccination (24).

Parental lack of confidence is not simply caused by poor health literacy. Pervasive online vaccine misinformation undermines public trust and intensifies vaccine hesitancy among parents (26). Therefore, vaccine safety can be guaranteed by strengthening quality inspection of HPV vaccines, and correct HPV vaccine knowledge can be disseminated to parents. In addition, at this stage, negative information about HPV vaccines spread in the problematic online environment has seriously damaged parents’ trust in HPV vaccines. We can carry out targeted professional vaccine knowledge training for medical staff, and official media can standardize the release of information and clarify false rumors, so as to improve parents’ confidence in HPV vaccines.

In addition, the results of this study show that subjective norm was positively associated with behavioral intention, and its effect is second only to attitude toward the behavior: the higher the score, the greater social support parents feel, and the higher their enthusiasm to vaccinate their children against HPV. Among all sources of social support, medical staff appeared to be a potentially important source of vaccination-related support than family members and social media, which can effectively improve parents’ willingness.

Nevertheless, differing from the routine counselling model of family physicians overseas, primary care facilities in China have not fully exerted their capacity for HPV-related health education. It is necessary to strengthen health education in medical institutions, implement regular HPV vaccine promotion, and publicize the importance of timely vaccination via diverse channels to improve awareness among medical personnel, patients and their families.

The negative correlation between perceived behavioral control and behavioral intention contradicts the classical hypothesis of the theory of planned behavior. This counter-intuitive result may be attributed to the operationalization of perceived behavioral control: the scale items predominantly captured behavioral barriers rather than facilitating conditions. Higher scores in this context reflected greater perceived obstacles to performing the behavior, which in turn suppressed behavioral intention, yielding a negative coefficient statistically.

Perceived behavioral control showed a negative association with behavioral intention, which indicates that the stronger the negative factors such as vaccination price, concerns about side effects, doubts about the quality of domestic vaccines, child’s age, perceived complexity of HPV treatment and distance to vaccination sites, the weaker parents’ behavioral intention to vaccinate their children. This indicates that improving attitudes alone may be insufficient to ensure conversion of willingness into vaccination uptake. Efforts should be made to improve the accessibility of vaccination services, alleviate excessive public concerns about adverse reactions, and strengthen parents’ perceived behavioral control. Drawing on practical experience of primary vaccination services in low and middle-income countries in recent years, more HPV vaccination sites are recommended to be established in grassroots institutions such as township health centres and community health service centres to expand vaccination channels. In addition, the professional capacity of grassroots vaccinators should be enhanced, and sustained public health education should be carried out to reduce parental vaccination concerns.

Against the background of the global strategy to eliminate cervical cancer, individual-level health education alone cannot bridge the intention-behavior gap. Successful experiences from high-income countries suggest that institutional policy guarantees are the fundamental measure to raise vaccination coverage (22). Several regions in China including Guangzhou and Shenzhen have launched pilot programmes providing free or subsidised HPV vaccination for eligible girls. Pilot data verify that policy interventions can significantly increase vaccination rates, yet prominent regional disparities still exist nationwide (27). In the future, China can gradually expand the coverage of free or subsidised HPV vaccination policies for eligible girls, build a multi-stakeholder mobilisation and reminder mechanism linking schools, families and medical institutions, and remove vaccination barriers systematically.

This study has several limitations. First, an online self-administered questionnaire was adopted in this survey, so researchers could not effectively control the respondents’ survey completion environment. In addition, most participants were from rural areas, while the proportion of urban residents was relatively low, leading to certain sampling bias. Affected by regional socioeconomic conditions, living habits and healthcare resource availability, the sample can only represent rural populations in the local region and lacks data support from urban residents and rural groups in other regions. Accordingly, the external validity of this study is limited. The findings can only be cautiously generalized to similar rural populations in the study area and should not be directly extended to the whole country or other populations with different characteristics. Future multi-centre studies are warranted to expand the sampling scope, enrol participants from urban and rural areas across diverse regions, and further verify the results of this study. Second, this is a cross-sectional study, and the results only represent parents’ vaccination willingness at a specific time point. With changes in the surrounding environment, vaccination policies and epidemiological trends of diseases, parents’ attitude toward the behavior, subjective norm and perceived behavioral control towards HPV vaccines will also change, so more representative survey research is needed for further verification and supplement in the future.

## 5 Conclusion

Parents of female primary and secondary school students in Heze City have a moderate willingness to vaccinate against HPV, but the actual vaccination rate is low. Based on their attitudes, subjective norms, and perceived behavioral control of the HPV vaccine, HPV vaccine popularization and education should be strengthened, medical staff and community guidance should be strengthened, vaccination services and policy support should be optimized, parents’ willingness to be vaccinated should be improved, and strategies should be developed to facilitate the conversion of vaccination willingness into actual vaccination uptake.

## Data Availability

The raw data supporting the conclusions of this article will be made available by the authors, without undue reservation.
