# Supplementary material for: Analysis of factors influencing HPV vaccination willingness of parents of students in China based on the theory of planned behavior
Source: Front Public Health. 2026 Sep 16;14:1898825. doi: 10.3389/fpubh.2026.1898825 (PMC13625759; doi:10.3389/fpubh.2026.1898825)
Supplement: Supplementary file 1 [file Supplementary_file_1.docx]

**Supplementary File 1. English Translation of the Questionnaire**

**Parental Survey on HPV Vaccination for Daughters**

Dear Parent or Guardian,

This is an anonymous academic survey for parents of primary and secondary school students in Heze City. The study aims to understand parents' knowledge of HPV vaccination for their children, vaccination willingness, and behavioral intention. The questionnaire takes approximately 8-10 minutes to complete. There are no right or wrong answers. All data will be used only for research and personal information will be kept confidential. Completion of the questionnaire indicates informed consent to participate. Please answer according to your actual views. Thank you for your support.

**Part I. Basic Information**

1. Your relationship to the child: 1) Father 2) Mother 3) Other guardian

2. Your daughter's school stage: 1) Primary school 2) Senior high school

3. Your daughter's age: ____ years

4. Your age: 1) <35 years 2) 35-45 years 3) >45 years

5. Your highest education level: 1) Primary school, junior high school, or below 2) Senior high school/technical secondary school 3) University or above

6. Monthly household income: 1) <RMB 5,000 2) RMB 5,000-10,000 3) >RMB 10,000

7. Place of residence: 1) Rural area 2) Urban area

8. Have you heard of HPV? 1) Never heard of it 2) Heard of it but do not understand it 3) Know a little about it 4) Fairly familiar with it

9. Has your daughter already received an HPV vaccine? 1) Yes 2) No

10. Your occupation: 1) Farming 2) Enterprise employee 3) Civil servant or technical professional

11. How busy is your daily work? 1) Very busy 2) Busy 5) Not busy

12. Type of family health insurance (multiple selections allowed): □ Basic medical insurance for urban and rural residents □ Basic medical insurance for urban employees □ Commercial medical insurance □ No health insurance

13. Has anyone in your family had cervical cancer or another reproductive tract tumor? (Family history of cancer) 1) Yes 2) No

**Part II. HPV Vaccine Knowledge and Vaccination Willingness (multiple selections allowed)**

14. Through which channels have you learned about the HPV vaccine? □ Short-video platforms/internet □ Television/newspapers □ Doctor/community health center □ School publicity □ Relatives and friends □ Other

15. Are you willing to have your child vaccinated? 1) Willing 2) Unwilling

**Part III. Theory of Planned Behavior Items (5-point Likert Scale)**

Response options for each item: Strongly disagree (1) Disagree (2) Neutral (3) Agree (4) Strongly agree (5)

**Attitude Toward the Behavior**

| **Item** | **Response options** |
| --- | --- |
| C1 | The HPV vaccine can effectively prevent HPV infection and cervical cancer. |
| C2 | My child needs to receive the HPV vaccine. |
| C3 | I would feel reassured after my child completes HPV vaccination. |
| C4 | HPV vaccination is beneficial for age-eligible people. |

**Subjective Norm**

| **Item** | **Response options** |
| --- | --- |
| C5 | If relatives and friends recommend it, I will have my child receive the HPV vaccine. |
| C6 | If a doctor recommends it, I will have my child receive the HPV vaccine. |
| C7 | If the government (community) recommends it, I will have my child receive the HPV vaccine. |

**Perceived Behavioral Control**

| **Item** | **Response options** |
| --- | --- |
| C8 | The complexity of HPV infection treatment will affect my decision about vaccination. |
| C9 | The high cost of the HPV vaccine will affect my decision. |
| C10 | Vaccine side effects will affect my decision. |
| C11 | The effectiveness of the HPV vaccine will affect my decision. |
| C12 | Whether my child's age is appropriate for vaccination will affect my decision. |
| C13 | Uncertainty about the quality of domestic HPV vaccines will affect my decision. |

**Behavioral Intention**

| **Item** | **Response options** |
| --- | --- |
| C14 | I will recommend the HPV vaccine to relatives and friends. |
| C15 | I am considering having my daughter receive the HPV vaccine. |
| C16 | At an appropriate time in the future, I will have my daughter receive the HPV vaccine. |

This concludes the questionnaire. Thank you again for taking the time to complete this survey.
